# Sex, Sterilization and Metabolic profiles Shape Aging Trajectories and Age-related Disease Risk in Dogs

**DOI:** 10.1093/geroni/igaf122.2885

**Published:** 2025-12-31

**Authors:** Filippo Artoni, Ben Harrison, Laura Corlin, Daniel Promislow

**Affiliations:** Jean Mayer USDA Human Nutrition Research Center on Aging, Boston, Massachusetts, United States; University of Washington, Seattle, Washington, United States; Tufts University, Medford, Massachusetts, United States; Jean Mayer USDA Human Nutrition Research Center on Aging, Boston, Massachusetts, United States

## Abstract

The metabolome is a highly conserved network of small molecules that plays a central role in cellular metabolism and signaling, yet its role in aging remains poorly understood. Here, we leverage the companion dog as a powerful translational model to investigate age-related metabolic changes and their relationship with disease. Dogs share a similar environment with humans, exhibit age-related multimorbidity, and have shorter lifespans, making them ideal for studying aging dynamics. Using machine-learning models trained on longitudinal data from the Dog Aging Project (DAP), we constructed a comprehensive model of the aging metabolome. Our dataset included metabolomics, blood chemistry, and complete blood counts from DAP’s Precision cohort (n = 976), along with demographic, health, and lifestyle data from DAP’s Pack cohort (>50,000). We identified metabolic shifts associated with aging and age-related conditions, including osteoarthritis (OA), where metabolites such as dimethylglycine and betaine were associated with disease risk. Additionally, large, giant, and mixed breeds, as well as sterilized dogs, exhibited an increased risk of OA, whereas no significant sex-based differences were observed. These findings provide new insights into the metabolic underpinnings of age-related diseases and highlight the influence of body size and sterilization status on disease risk.

